# IgG4 autoantibodies and autoantigens in the context of IgG4-autoimmune disease and IgG4-related disease

**DOI:** 10.3389/fimmu.2024.1272084

**Published:** 2024-02-16

**Authors:** Rodrigo V. Motta, Emma L. Culver

**Affiliations:** ^1^ Translational Gastroenterology and Liver Unit, Nuffield Department of Medicine, University of Oxford, Oxford, United Kingdom; ^2^ Department of Gastroenterology and Hepatology, Oxford University Hospitals NHS Foundation Trust, Oxford, United Kingdom

**Keywords:** IgG4, IgG4-RD, autoantibody, antigen, autoimmunity

## Abstract

Immunoglobulins are an essential part of the humoral immune response. IgG4 antibodies are the least prevalent subclass and have unique structural and functional properties. In this review, we discuss IgG4 class switch and B cell production. We review the importance of IgG4 antibodies in the context of allergic responses, helminth infections and malignancy. We discuss their anti-inflammatory and tolerogenic effects in allergen-specific immunotherapy, and ability to evade the immune system in parasitic infection and tumour cells. We then focus on the role of IgG4 autoantibodies and autoantigens in IgG4-autoimmune diseases and IgG4-related disease, highlighting important parallels and differences between them. In IgG4-autoimmune diseases, pathogenesis is based on a direct role of IgG4 antibodies binding to self-antigens and disturbing homeostasis. In IgG4-related disease, where affected organs are infiltrated with IgG4-expressing plasma cells, IgG4 antibodies may also directly target a number of self-antigens or be overexpressed as an epiphenomenon of the disease. These antigen-driven processes require critical T and B cell interaction. Lastly, we explore the current gaps in our knowledge and how these may be addressed.

## 1 Introduction

Antibodies are the fundamental component of humoral immune responses, also known as antibody-dependent responses. These molecules can recognise and neutralise pathogens, either by binding to molecular antigens and directly preventing their pathogenic effect, or by opsonising these pathogens and triggering effector functions, such as the complement system, antibody-dependent cell-mediated cytotoxicity and antibody-dependent cellular phagocytosis.

Five different classes of antibodies, or immunoglobulins (Ig) are found in humans, namely IgM, IgD, IgA, IgE and IgG. IgM and IgD are involved in primary adaptive humoral immunity and can be found in mature naïve B cells (1), which undergo specialisation and a class switch towards long lived B cells and plasmablasts that produce IgA, IgE and IgG (2, 3). IgA is mainly involved in mucosal humoral immunity. IgE is one of the main components of allergic reactions. IgG is part of several different processes both in health and disease.

IgG has four subclasses (i.e., IgG1, IgG2, IgG3 and IgG4) which form part of the immune response. Under normal conditions, IgG1-3 can fixate complement and opsonise pathogens, whilst IgG4 only activates complement under special circumstances, at high antibody and antigen concentrations. IgG and its subclasses are involved in autoimmune processes, such as rheumatoid arthritis, systemic lupus erythematous, ANCA-associated vasculitides, as well as IgG4-autoimmune diseases (IgG4-AID) and IgG4-related disease (IgG4-RD).

In this review, we describe the structure and function of IgG4 antibodies, discuss the role of this class in allergy, helminth infections, malignancies, and autoimmune diseases. A greater emphasis is placed on IgG4-related disease and the role of IgG4 antibodies in the pathogenesis of the disease.

## 2 Structure and function of IgG4 antibodies

**Figure 1:**
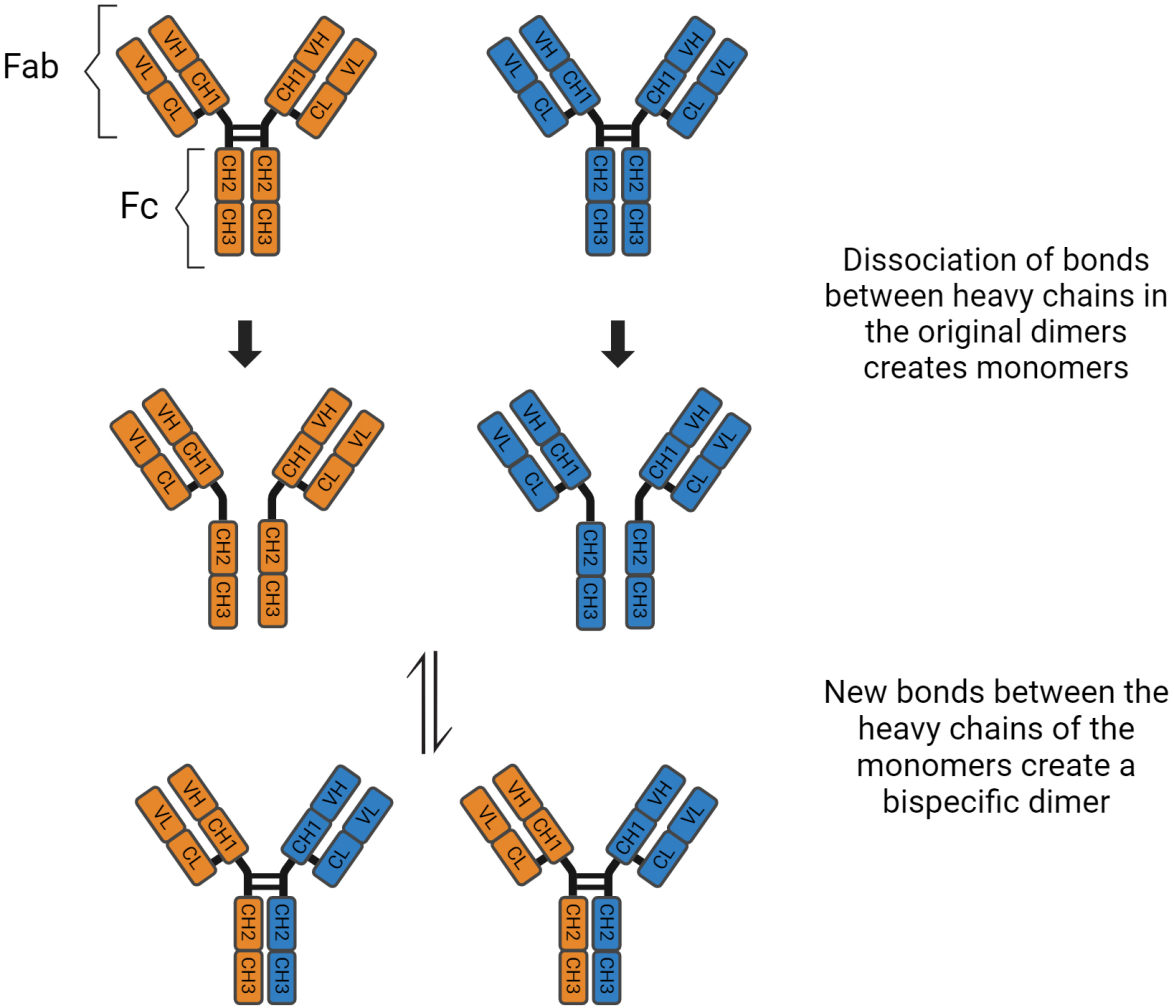
Fab arm exchange in IgG4 antibodies.

Immunoglobulin G has four subclasses, with IgG4 representing up to 5% of the total IgG concentration (4). Their structure is based on 2 heavy and 2 light chains bound together by disulphide bridges. There are interactions between the light and heavy chains, as well as a connection between the two heavy chains at the hinge region. This region is important because it gives structural flexibility to the molecule. Both the light and heavy chains have antigen-binding sites, the variable region (V_L_ and V_H_), and areas responsible for the effector function of the antibodies, called constant region (C_L_ and C_H_1, C_H_2, C_H_3, C_H_4). Furthermore, these molecules can be divided by function, the “Fragment, antigen binding” (Fab) region and the “Fragment, crystallised” (Fc) region, which is responsible for the effector function (5). In the CH2 domain, a switch of proline to serine at position 228 in the hinge of IgG4 facilitates Fab-arm exchange (**Figure 1**). Schuurman, Aalberse and colleagues observed that this phenomenon is marked by the dissociation of the two heavy chains and recombination of two random IgG4 monomers (heavy and light chains) to form a bispecific dimer (6). Even though this new molecule might recognise two different antigens, it is functionally monovalent, and cannot form large immune complexes (7–9). The Fc portion in IgG4 antibodies have a high affinity for antigens and FcγIIb receptors but low affinity for Fcγ stimulatory receptors and cannot tipically activate the classical complement pathway (10). These characteristics hinder Fc mediated effector functions and prevent further sensitisation of the immune system (8, 11–13). There are, however, situations where IgG4 molecules can activate the complement pathway. The first is via recruiting mannose-binding lectins and activating the lectin pathway, which has been observed in anti-PLA2R membranous nephropathy (14). The second is through mutations of the Fc region, enabling these molecules to form hexamers and bind to C1q (15). These molecules can also activate the complement system using the classical pathway when there is high antigen density and high antibody concentration (16). Finally, Oskam, Rispens and collaborators showed that in opposition to previous evidence, the heavy chains of IgG4 molecules can dissociate and interact with the Fc region of other IgGs. It is not clear whether this mechanism plays a role in the pathogenesis of autoimmune diseases, but it modulates the affinity of IgG1 and IgG2 for C1q (17). Hence, IgG4 has anti-inflammatory properties, and it is involved both in health, such as the development of tolerance in allergies (18), as well as disease, such as curbing immune responses against helminth infections (19) (**Table 1**).

**Table 1:** IgG4 antibodies: structural characteristics and functional consequences.

Molecule structure	Function
**Change of proline (IgG1) to serine (IgG4) at position 228 in the core hinge region**	Asymmetry in the structure with different Fab arms. The final antibody is functionally monovalent. **IgG4 cannot bind to two different antigens neither form large immune complexes.**
**CH2 domain amino acid substitutions - L234F and P331S**	Low-affinity to FcγRIIa and FcγRIIIa. **Weak ability to bind to FcγRI compared with IgG1**
**CH2 domain amino acid substitution - P331S**	Negligible binding to the C1q protein complex. **Cannot trigger the classical complement pathway**
**Constant domain links with Fc portion of other IgG molecules**	Compete with other antibodies and impairs IgG1 ability to activate complement or form immune complexes. **Anti-inflammatory function**

In the function column, the bold values represent the functional outcomes.

## 3 Class switch recombination and somatic hypermutation

When exposed to antigens, naïve B cells become antibody secreting B cells via the extrafollicular and follicular pathways, which are complementary. The initial response to antigens is predominantly driven by the extrafollicular path (20, 21). It involves T follicular helper (Tfh) cells driving the differentiation of naïve B cells into short lived plasmablasts, which will maintain an immune response for up to a week after the trigger (20, 22).

To create an immune response based on IgG4, B cells must undergo class switch recombination (CSR) and somatic hypermutation (SHM) (11, 23, 24). These processes are hallmarks of follicular responses, which take part in germinal centres (GC) of lymph nodes and the spleen. In the follicular pathway, antibody secreting B cells develop high affinity and specificity and become either memory B cells or long-lived plasma cells (LLPC). The GCs are divided in dark and light zones, with functional differences. The dark zone promotes proliferation of B cells, as well as CSR and SHM (25). CSR is marked by deletional recombination in the heavy chain gene that leads to a change in Ig class, thus changing effector functions of the antibodies, while preserving the affinity for specific antigens. Given its mechanism, switch in Ig class follows a sequential pattern from IgM/IgD to IgG3, IgG1 and IgA1, called proximal classes. A secondary switch then gives rise to distal classes such as IgG2, IgG4 and IgA2 (23, 26, 27) and has been attributed to stimuli from T helper 2 (Th2) cells. Interleukin (IL) 4 and IL-13 are responsible for skewing the antibody production to IgG4 and stimuli involving IL-10 and/or IL-21 lead to selection of IgG1 antibodies (28). After these clones have developed high-affinity and specificity, they migrate into the light zone. There, they will undergo a selection according to interactions with Tfh cells and follicular dendritic cells (29). Once the highly specific clones are selected, they leave the GC and become LLPC or migrate to the bone marrow as memory B cells.

## 4 IgG4 in allergic reactions

IgG4 antibodies have an important role in the development of tolerance in atopic patients. It has been extensively shown that beekeepers develop an IgG4-based response to bee venom associated with reduction of symptoms after exposure to the toxin (30, 31), and the same event has been described in other cases of occupational exposure (32, 33). Furthermore, allergen-specific immunotherapy is based on the development of IgG4 antibodies against allergens through regular and incremental exposure to allergenic antigens (34). IgG4 competitively binds to allergens, thus hindering the formation of IgE-antigen immune complexes (35, 36). Furthermore, it also binds to FcγRIIb, an inhibitory Fcγ receptor, preventing the degranulation of mast cells and the cascade that would lead to allergic responses (37–39).

On the other hand, IgG4 antibodies are also involved in the maintenance of the disease in cases of eosinophilic oesophagitis (EoE) and eosinophilic chronic rhinosinusitis (40–42). The most robust evidence that this class is involved in disease comes from studies associating it with symptoms in patients with EoE. During food avoidance tests, patients referred less symptoms and biopsies showed reduction of IgG4 deposition (43). In addition, histological levels of IgG4 were found to be significantly lower during remission when compared to samples collected during disease activity (44). For a detailed review on the topic, please see (45).

## 5 IgG4 in helminthic infections

Helminthic infections are another scenario where IgG4 antibodies show their tolerogenic effects. Patients with acute parasitic infection usually show a balanced Th1/Th2 immune response, with a predominance of IFN-γ over IL-10, and a higher count of peripheral eosinophils (46). If the pathogen fails to evade the host response, this leads to parasite clearance and termination of the infection.

Individuals with chronic infection, however, show an immune response with a Th2 profile, where IL-10 plays a critical modulatory role in skewing antibody production towards IgG4 (46). A response based on parasite-specific IgG4 antibodies, then, dampens IgE-mediated immune response and perpetuates the infection. Asymptomatic patients show a restricted response to parasite antigens, while those with severe chronic disease have lower levels of serum IgG4 antibodies and show signs of organ damage (46). Studies also show that individuals with a high parasite burden have higher levels of parasite-specific IgG4 than patients with a lower parasite burden (47, 48). The immunomodulatory effects of helminthic infections are also seen in the response to allergens, where patients with chronic parasitic infection show lower levels of IgE-reactivity to dust antigens (49).

## 6 IgG4 in malignancies

Antitumour humoral responses can prevent malignancies from developing further and even annihilate them by triggering humoral and cell-mediated immune responses. Some neoplasms induce class switch of anti-tumour antibodies to the IgG4 class, leading to immune escape and IgG4-mediated tumour-tolerogenic responses (50). Melanoma and pancreatic ductal adenocarcinoma cells induce a modified Th2 immune response around their microenvironment, triggering B cells to undergo antibody class switch towards IgG4 (51, 52). These antibodies will then competitively bind to cancer antigens and damp anti-tumour immune responses. Higher serum levels of IgG4 are associated with fewer cytotoxic T cells (53) and with a worse prognosis in patients with cholangiocarcinoma and melanoma (50, 54).

A meta-analysis reported that patients with IgG4-RD have an increased risk of clonal B cell lymphoma and pancreatic malignancy when compared with a matched general population (55). The mechanisms driving this increased risk remain speculative and include 1) chronic inflammation-induced carcinogenesis; 2) IgG4-related tumour-induced immune escape; 3) paraneoplastic phenomenon, as most malignancies detected distant from the site of disease activity. The class switch towards IgG4 induced by some malignancies may also play a role in the initiation of IgG4-RD. K-ras codon gene mutations associated with gastrointestinal cancer have been reported in patients with IgG4-related pancreatitis (56). These mutations are also associated with cellular transformation and genetic instability (57, 58).

## 7 IgG4 in IgG4-autoimmune diseases

Huijbers et al. were the first to collectively describe IgG4 autoimmune diseases (IgG4-AID) as a spectrum of conditions characterised by autoantibody responses against a known antigen (59). Evidence shows that these diseases are caused by IgG4 antibodies recognising autoantigens, although not all antigens have been validated. The diagnosis of IgG4-AID depends on high level of suspicion and identification of IgG4 autoantibodies against the antigen(s) specific to each disease (60). Koneczny proposed a classification system based on evidence of antibodies against extracellular antigens, and direct pathogenic mechanism of IgG4 antibodies observed *in vitro* and validated in animal models through passive transfer (61). Although 29 antigens have been described in IgG4-AID (**Table 2**), only the six antigens have met all three Koneczny criteria of IgG4 pathogenicity (61). For a thorough review on IgG4-AID and autoantigens involved in these disorders, please read (62).

**Table 2:** Comparison between antigens, symptoms, animal models, HLA associations and responsiveness to immunosuppression between IgG4-AID and IgG4-RD.

Antigen	Localisation of antigen	Disease	Symptoms	Organ affected	Evidence of passive transfer	Evidence through active immunisation	Ig type	Response to immunosuppressants	HLA associations
Carbonic anhydrase II	Cytoplasm	IgG4-RD	Pseudotumours, fibrosis. Varies according to organ affected.	Pancreas, bile ducts, sallivary and lacrimal glands, kidneys, retroperitoneum, aorta, orbits	No	Yes	IgG4	Yes	HLA-DQB1*0401, HLA-DRB1*0405, HLA-DRB1*16, FCGR2B
Plasminogen binding protein type A/UBR2	Cytoplasm and nucleus				No	No	Total IgG		
Lactoferrin	Pancreatic juice				No	No	Total IgG		
Pancreatic secretory trypsinogen inhibitor (SPINK1)	Pancreatic juice				No	No	Total IgG		
Trypsinogens	Pancreatic juice				No	No	Total IgG		
Annexin A11	Cytoplasm, cell membrane				Yes	No	IgG1 and IgG4		
Laminin 511-E8	Extracellular matrix				Yes	Yes	IgG1 and IgG4		
Galectin 3	Extracellular matrix, cytosol and nucleus				No	No	IgG4 and IgE		
Prohibitin	Nucleus, cytoplasm and mitochondria				No	No	IgG4		
Desmoglein 1	Cell membrane	Pemphigous foliaceous	Blistering lesions	Skin	Yes	Yes	IgG4 and IgG1	Yes	HLA-DRB1*0404, *1402, *1406, or *0102
Desmoglein 3	Cell membrane	Pemphigus vulgaris	Blistering lesions	Skin	Yes	No	IgG4 and IgG1	Yes	HLA-DRB1*14, HLA-DRB1*04:02, HLA-B*38, HLA-B*55
Muscle-specific kinase	Cell membrane	MuSK myasthenia gravis	Weakness and atrophy of neck, tongue, shoulder and bulbar muscles.	Muscles (Motoneural junction)	Yes	Yes	IgG4	Yes	HLA-DQB1*05, HLA-DRB1*13, HLA-DRB1*16
Contactin 1	Cell membrane	Chronic inflammatory demyelinating polyradiculoneuropathy	Subacute neuropathy, sensory ataxia and tremor.	CNS and PNS	Yes	No	IgG4	Variable	HLA-DQB1*02
Neurofascin 155	Cell membrane				Yes	No	IgG4	Variable	
CASPR1	Cell membrane				No	No	IgG4	Variable	
Neurofascin 140/186	Cell membrane				No	No	IgG4	Variable	
ADAMTS13	Blood circulation	Thrombotic thrombocytopenic purpura	Thrombotic events	Blood	Yes	No	IgG1 and IgG4	Yes	HLA-DRB1*11, HLA-DRB1*08:03
CASPR2	Cell membrane	Isaac’s syndrome, Morvan’s syndrome, limbic encephalitis	Epilepsy, behavioural and mental abnormalities, autonotmic instability, motor and sensory neuropathy.	CNS and PNS	Yes***	No	IgG4	Variable	HLA-DRB1*11:01
LGI1	Cell membrane	LGI1-associated limbic encephalitis, Isaac’s syndrome	Epilepsy, behavioural and mental abnormalities, seizures, dystonia, sensory and motor neuropathy.	CNS and PNS	Yes***	No	IgG4	Variable	HLA-DRB1*07:01
PLA2R	Cell membrane	Membranous nephropathy	Oedema, fatigue, changes in urine	Kidneys	Yes	Yes****	IgG4	Yes	HLA-DQA1, HLA-DRB1*1501, HLA-DRB1*0301
THSD7A	Cell membrane				Yes	No	IgG4	Yes	
Alfa-enolase	Cell membrane				No	No	IgG4 and IgG1	Yes	
Superoxide dismutase 2 (SOD2)	Mitochondria				No	No	IgG4 and IgG1	Yes	
Aldose reductase	Cytoplasm				No	No	IgG4 and IgG1	Yes	
GPIHBP1	Cell membrane	GPIHBP1 autoantibody syndrome	Severe hypertriglyceridemia	Blood	No	No	IgG4	Needs more studies	
Laminin 332	Cell membrane	Mucous membrane pemphigoid	Blistering mucosal lesions, mainly in the oral cavity and conjunctivae	Mucosa	Yes	No	IgG4	Yes	HLA-DQB1*0301
Type IV Collagen	Extracellular matrix	Goodpasture syndrome, epidermolysis bullosa acquisita (EBA)	Haemoptysis, changes in urine/blistering lesions	Lungs and kidneys, skin	Yes	Yes	IgG1 and IgG2	Yes	HLA-DRB1*1501/HLA-DR15, HLA-DRB1*15:03
IgLON5	Extracellular matrix	IgLON5 parasomnia	gait instability, non-REM and REM parasomnia, cognitive decline, movement disorders.	CNS	No	No	IgG1	Variable	HLA-DRB1*10:01-DQB1*05:01
DPPX	Cell membrane	Anti-DPPX encephalitis	Weight loss, gastrointestinal symptoms, cognitive-mental dysfunction, tremor, seizures, autonomic dysfunction.	CNS	No	No	IgG4 and IgG1	Variable	
ANCA	Cytoplasm	ANCA-related vasculitides	Fatigue, haemoptysis, muscle pain, changes in urine	Blood vessels, kidneys	Yes	Yes	IgG1, IgG3, IgG4	Yes	HLA-DRB1*04, HLA-DRB1*07, HLA-DRB1*09, HLA-DRB3, HLA-DRB1*13, HLA-DQ
Bullous pemphigoid antigen 180	Cell membrane	Bullous pemphigoid Bullous pemphigoid	Blistering lesions Blistering lesions	Skin	Yes	Yes	IgG1	Yes Yes	HLA-DQB1*0301 HLA-DQB1*0301
Bullous pemphigoid antigen 230	Cytoplasm			Skin	Yes	Yes	IgG1		
IFN 1, IL-17A, IL-22	Extracellular matrix	Autoimmune polyendocrinopathy-candidiasis-ectodermal dystrophy	Chronic candidiasis, hypothyroidism, hypogonadism	Skin, mucosa, endocrine organs, lungs, bowels	No	No	IgG1 and IgG4	Yes*/**	
P200 (laminin gamma1)	Cell membrane	Anti-p200 pemphigoid	Blistering lesions	Skin	No	No	IgG4	Needs more studies	

*Only the autoimmune manifestations respond to immunosuppressants.

** Ruxolitinib might be effective in the treatment of chronic mucocutaneous candidiasis in patients with STAT1 gain-of-function mutations.

***Did not trigger all the symptoms observed in patients.

****Knock-in PLA2R mice spontaneously developed MN.

### 7.1 Desmoglein 1 (Dsg1) and 3 (Dsg3):

Desmoglein 1 and 3 are transmembrane proteins found in keratinocytes. Anti-Dsg1 IgG4 is responsible for the development of pemphigus foliaceous (PF), while anti-Dsg3 IgG4 antibodies, and in some cases anti-Dsg1 IgG4, cause pemphigus vulgaris. These antibodies target epitopes in EC1 and EC2 domains, including in fogo selvagem (FS), an endemic PF specific to Brazil (63), and in the endemic PF found in Tunisia (64). Qian and Peng observed a cross-link between anti-LJM11, a protein present in the saliva of sand flies (*L. longipalpis*), and anti-Dsg1 antibodies in patients with FS and healthy controls from the same region (65, 66). The difference being that antibodies from the healthy population recognised the EC5 domain of Dsg1 rather than EC1 and EC2 (63, 65–67). Studies investigating the Tunisian population found that endemic PF patients and healthy controls from the same area had anti-Dsg1 IgG and demonstrated a cross-link between these antibodies and the salivary extract from *P. papatasi* (68–70). The antibodies in healthy Tunisians, instead of targeting the epitopes involved in endemic PF, bound to epitopes in the EC3, EC4 and EC5 of Dsg1 (64) and were mostly IgG1, IgG2 and IgG3 (71)

### 7.2 Muscle-specific kinase (MuSK):

MuSK is a tyrosine kinase involved in the transduction of electrical signals at the neuromuscular junction and is involved in MuSK myasthenia gravis (MG) (72). Anti-MuSK IgG4 antibodies often target the Ig-like 1 domain of the protein (73–75), which has important functional implications. This region contains the site where lipoprotein receptor-related protein 4 (Lrp4) binds to MuSK and activates the kinase, which causes clustering of acetylcholine receptors (AchR) at the synapse. By competitively binding to that domain, IgG4 antibodies block Lrp4-dependent activation of MuSK (76, 77), thus impairing migration of AchR to the neuromuscular junction. Other IgG subclasses might also be involved in MuSK MG. Huijbers et al. observed that valency affects the outcomes of antibody binding to MuSK and may, in fact, lead to activation of the kinase (78). Furthermore, patients with MuSK-MG have higher levels of IgG4, but they also have higher levels of IgG1 when compared to controls, which does not corroborate the theory that these patients have overall responses biased towards IgG4 (79).

### 7.3 Contactin 1 (CNTN1):

Contactin 1 binds with CNTN1-associated protein 1 (Caspr1) and neurofascin 155 (NF155), forming an axo-glial complex that is involved in paranode architecture and maintenance of myelin in axons (80). Chronic inflammatory demyelinating polyradiculoneuropathy (CIDP) is a IgG4-AID, and one of the possible antigens in this disorder is CNTN1. Anti-CNTN1 IgG4 antibodies recognise the IgC2 domains of CNTN1 and block its interaction with NF155, which leads to impaired cell aggregation and changes in paranodal architecture (81). Patients may present with other autoantibodies and have different clinical syndromes. Those presenting antibodies against neurofascin 186 (NF186), for instance, may present with renal impairment given both CNTN1 and NF186 are found in podocytes (62, 82).

### 7.4 Neurofascin 155 (NF155):

Neurofascin 155 is expressed by myelinating glial cells and, alongside Caspr1 and CNTN1, is part of the septate-like junction in paranodes found both in the central and peripherical nervous systems. Animal studies have confirmed the pathogenicity of anti-NF155 antibodies. Manso and colleagues demonstrated that intrathecal infusion of IgG4 from anti-NF155 CIDP patients induced similar symptoms in previously healthy mice (83). Early studies identified that these antibodies recognise the Fn3 and Fn4 domains of NF155, which does not participate in the interaction with CNTN1, and suggested a possible blocking mechanism that would cause the clinical presentation (84). Nevertheless, Manso et al. found that anti-NF155 IgG4 is associated with aggregation of the antigen and induces its depletion. They could not determine which mechanism was responsible for the depletion of surface NF155 and suggested that it might have to do with proteolysis of the antigen or molecular instability (83, 85).

### 7.5 ADAMTS13:

ADAM metallopeptidase with thrombospondin type 1 motif 13 (ADAMTS13) is a protease found in the blood circulation. It is responsible for the proteolytic cleavage of the multimeric form of von Willebrand factor (vWf) and ensuring normal haemostasis (86). Anti-ADAMTS13 IgG4 antibodies and vWf recognise the spacer-domain of the antigen, thus creating a competition for binding with ADAMTS13 (87). Autoimmune thrombotic thrombocytopenic purpura (TTP) is marked by an initial response based on IgG1 antibodies against ADAMTS13. The chronic antigenic stimulation then promotes class switch towards IgG4, which perpetuates the disease (88). The accumulation of vWf causes platelet aggregation and formation of microthrombi, which leads to the characteristic phenotype of microangiopathic haemolytic anaemia.

## 8 IgG4 in IgG4-related disease

IgG4-related disease (IgG4-RD) is a chronic relapsing immune-mediated fibro-inflammatory disorder and its hallmark is the presence of IgG4 antibodies in the sites affected (89). The diverse organ involvement in this disease is linked by a unique histopathology; a lymphoplasmacytic infiltrate rich in IgG4+ plasma blasts and CD4^+^ T cells, storiform fibrosis, and obliterative phlebitis (90).

There are currently three theories on the role of IgG4 antibodies in IgG4-RD: 1) IgG4 antibodies directly cause the disease by targeting self-antigens; 2) IgG4-RD patients have an immune response inherently biased towards production of IgG4 antibodies to any stimulus; 3) IgG4 is only present to modulate an immune response based on different pathways. It continues to be elusive whether the overexpression of these antibodies is an epiphenomenon of the inflammatory reaction or has a causal role in disease pathology.

### 8.1 Do IgG4 antibodies directly cause disease by targeting self-antigens?

Next-generation sequencing studies yield evidence to support that the pathogenesis of IgG4-RD is an antigen-driven process mediated by B and T cell interactions (91). Circulating B cells are predominantly IgG4^+^ B cells with extensive V-region mutations affecting both hypervariable and framework regions, suggesting signs of antibody maturation (91, 92). Mattoo et al. observed expansion of CD19^+^CD27^+^CD20^-^CD38^hi^ plasmablasts, which are oligoclonally restricted as shown by analysis of immunoglobulin heavy V and J regions (92). Naïve B cells then undergo *de novo* recruitment during disease relapse. In patients who have a disease flare after treatment with rituximab, activated B cells and plasmablasts have increased somatic hypermutation when compared to before B cell depleting therapy, suggesting a possible driver for the autoreactive pathogenic process (92).

Differences in B cell phenotypes may be clinically important, as demonstrated by Li and colleagues (93). Patients with active IgG4-RD had increased plasmablasts (CD19^+^CD24^-^CD38^hi^, CD19^+^CD27^hi^CD38^hi^, and CD19^+^IgD^-^CD38^hi^) and reduced numbers of CD19^+^ B cells, IgD^+^CD38^+/-^ naïve B cells and CD24^hi^CD38^hi^ B regulatory cells (Breg) when compared to controls (93). Cluster analysis revealed that patients with a high count of plasmablast and memory B cells, low count of naïve B cells and Bregs had a higher disease burden and were predominantly male (93). This highlights the importance of antigen-specific LLPC in the pathogenesis of IgG4-RD. Whilst Rituximab (anti-CD20) reduces the number of short-lived B cells expressing CD20, memory B cells and LLPC lack this surface protein and can evade depletion, contributing to the relapse of disease.

Analysis of IgG4-RD lesions showed that tissue CD4^+^ T cells are clonally expanded with a cytolytic phenotype, expressing granzyme A, perforin and SLAMF7^+^ (SLAM family member 7) (94), and secreting IL-1β, TGF-β and IFN-γ, cytokines associated with fibrosis (94, 95). Drugs that deplete B cells lead to profound clinical remission and significantly reduce serum plasmablasts and CD4^+^ cytotoxic cell populations. Therefore, the excellent response to B cell depletion support that these cells are involved in the pathophysiology of IgG4-RD (93, 96, 97). Overall, this evidence suggests that T-dependent B cell activation is essential in the pathogenesis of IgG4-RD. For a thorough review of the roles of different T cells in IgG4-RD, please see (91).

HLA-mediated antigen presentation occurs in a number of autoimmune diseases, such as DRB1*15 in multiple sclerosis, DRB1*04 in rheumatoid arthritis and DQB1*02 and DQB1*03 in coeliac disease. A genome-wide association study in 857 Japanese IgG4-RD patients suggested that the HLA-DRB1*04:05 allele was important in the development of IgG4-RD (98). Indeed, mice expressing this risk allele develop autoimmune pancreatitis (99). This allele is also associated with type 1 diabetes and Crohn’s disease (100, 101). Furthermore, the single nucleotide polymorphism (SNP) rs134097 found in FcγRIIb was strongly associated with IgG4-RD, as well as with two phenotypes of the disease (pancreato-biliary and Mikulicz), number of swollen organs and serum IgG4 level at diagnosis. This SNP might play a critical role in IgG4-RD because its locus directly impacts on the expression of FcyRIIb, a receptor involved in the elimination of self-reactive B cells.

#### 8.1.1 Autoantigens in IgG4-RD

Many studies suggest a role of autoantigens in patients with IgG4-RD. Neonatal mice that received passive transfer of purified IgG1 and IgG4 from patients with active IgG4-RD showed evidence of damage to salivary glands and the pancreas (102). Several autoantibodies have been identified in the context of IgG4-related autoimmune pancreatitis, such as lactoferrin, carbonic anhydrase, pancreas secretory trypsin inhibitor, amylase-alpha, heat shock protein and plasminogen-binding protein. However, many of these lacked sensitivity and specificity for those with other organ manifestations (103).

##### 8.1.1.1 *Helicobacter pylori* antigens

Human carbonic anhydrase II (CAII) is an enzyme found in the cytoplasm of pancreatic ductal epithelial cells, as well as in kidney tubules, gallbladder and glial cells. Given the discovery of antibodies against *H. pylori* in the sera of patients with IgG4-related pancreatitis and Sjogren’s syndrome (104–106), it was proposed that *H. pylori* infection could be driving the disease through molecular mimicry with CAII (107). Frulloni and colleagues identified that, in fact, immunoglobulins from IgG4-RD patients targeted plasminogen-binding protein type A (PBP), also an antigen found in *H. pylori*. This protein shares sequence homology with a human protein, ubiquitin protein ligase E3 component n-recognin 2 (UBR2). The group proceeded to show that 19 (95%) of their patients with IgG4-related pancreatitis had antibodies against PBP and similar results were replicated with UBR2 (108). A large study by Culver et al., however, did not replicate the findings. Among 69 IgG4-RD patients and 51 controls with autoimmune or inflammatory diseases, authors found similar T and B cell reactivity against PBP between groups (109). Jesnowski and colleagues investigated the presence of conserved sequences of H. pylori in the pancreatic tissue and juice of patients with IgG4-related pancreatitis and pancreatic cancer via nested PCR and couldn’t identify any signal of H. pylori DNA (110), corroborating previous findings that the bacterium is unlikely to be directly involved in the pathogenesis of IgG4-related pancreatitis.

##### 8.1.1.2 Acinar antigens

Lactoferrin (LF), pancreatic secretory trypsinogen inhibitor (SPINK1) and trypsinogens have been identified as possible antigens in IgG4-related pancreatitis (111, 112) and antibodies against the last two proteins have been reported in different cohorts of patients with the disease. Löhr and colleagues showed that patients with chronic IgG4-related pancreatitis have a severely reduced population of acinar cells in histopathological studies. The presence of antibodies against trypsinogens was replicated in an animal model of the disease, thus corroborating this finding (112). Nevertheless, they could not differentiate between subtypes of autoimmune pancreatitis and could not explain why acinar cells resume production of digestive enzymes during remission of IgG4-related pancreatitis.

##### 8.1.1.3 Annexin A11

Annexin A11 is part of a family of calcium-dependent phospholipid-binding proteins. It is found in the cytosol of cholangiocytes, pancreatic duct cells and islands of Langerhans as well as other tissues. Hubers and colleagues (113) identified IgG4 antibodies against annexin A11 in 9 (18%) patients with IgG4-related pancreatitis and cholangitis, whilst not in controls with other pancreatobiliary diseases, including malignancies. Reinforcing this finding, the location where this antigen was identified corresponded to the pattern of injury in pancreatobiliary disease (114). It was also found in patients with IgG4-related salivary involvement, suggesting that annexin A11 is not specific to pancreatobiliary involvement.

The ‘biliary bicarbonate umbrella’ is responsible for protecting human cholangiocytes from hydrophobic bile acid influx (115). Herta et al. reported that annexin A11 is necessary so that this defensive mechanism can be adequate. Antibodies against annexin A11 inhibited the function of this self-antigen, thus the authors speculate that this may impair the biliary bicarbonate umbrella and facilitate bile duct damage in patients with IgG4-related cholangitis (116).

Lastly, Hubers et al. demonstrated that IgG4 antibodies isolated from patients with IgG4-RD compete with IgG1 for binding to annexin A11, thus reinforcing that the first has an anti-inflammatory role in the disease pathogenesis (113). These results have been replicated in the passive transfer experiment with neonatal mice, where IgG from IgG4-RD patients induced a similar pattern of pancreatic and salivary lesion. Shiokawa and colleagues observed that both IgG1 and IgG4 antibodies could trigger the injury. The first, however, had its activity dampened by simultaneous injection of IgG4 antibodies (117).

##### 8.1.1.4 Laminin 511-E8

Laminin is a component of the extracellular matrix (ECM) of tissues, and integrin alfa-6-beta-4 is a cellular adhesion molecule that binds to laminins in the ECM. A Japanese group identified antibodies against laminin 511-E8 in 51% (26/51) of patients with IgG4-related pancreatitis and in only 1.6% of healthy volunteers (118). Sixteen percent (4/25) of those who did not have antibodies to laminin, had antibodies against its ligand, integrin alpha-6-beta-1.

Passive transfer and active immunisation animal models confirmed that mice that received human sera from patients with IgG4-related pancreatitis and those immunized with human laminin 511-E8 developed an immune response responsible for injury in pancreatic tissue and salivary glands in the same pattern as observed in IgG4-RD (119). Regardless of serum concentration of IgG4, half of these patients had specific IgG1 antibodies whilst only one patient had IgG4 against laminin 511-E8. Furthermore, only pancreatic and salivary gland injury was demonstrated.

Shiokawa observed that anti-laminin 511-E8 antibodies decreased upon treatment with glucocorticoids in an equivalent manner as other biomarkers of activity (119) (e.g., serum IgG4 concentration and pancreatic imaging). These results have not been validated externally. In contrast, In a US IgG4-RD cohort, the positivity rate for laminin 511-E8 antibody was similar between IgG4-RD (7%), disease controls, and healthy volunteers (118).

##### 8.1.1.5 Galectin-3

Galectin-3 is a cytoplasmatic b-galactoside-binding lectin identified in systemic fibroproliferative conditions, such as systemic sclerosis and pulmonary fibrosis (120). In a US cohort of 121 IgG4-RD patients, using immunoaffinity chromatography and mass spectrometry of plasmablast clones, 34 (28%) were positive for IgG4-specific anti-galectin-3 antibody while almost none of the 45 disease controls with interstitial pulmonary fibrosis and 50 healthy volunteers showed similar results (121). Furthermore, IgE-specific anti-galectin-3 antibodies were also detected but other IgG subclasses had little to no reactivity among the participants (121).

Perugino et al. used a galectin level threshold above 10.25ng/mL, which is independently associated with all-cause mortality in systemic sclerosis (122), to divide their cohort into two groups; those with higher levels had 64% positivity for IgG4 anti-galectin-3 antibodies, whilst those with lower levels had 23% positivity (121). Moreover, the authors reported a correlation between the presence of these antibodies and lymphadenopathy in IgG4-RD, as well as a trend between higher serum concentration of galectin-3 and increased IgG4-RD Responder Index of disease activity, number of organs and multi-organ involvement.

A Japanese group used proteomic analysis to demonstrate a 13-fold increased expression of galectin-3 in the pancreas of patients with IgG4-related pancreatitis compared with heathy pancreatic tissue (123). Galectin-3 is expressed by different cell lines in affected organs in IgG4-RD, including pancreas, bile ducts, salivary glands, kidney, lung, aorta and retroperitoneum, supporting a role in tissue fibrosis in IgG4-RD (124).

##### 8.1.1.6 Prohibitin

Prohibitins are ubiquitously expressed and are important in critical cell processes. Prohibitin 1 is involved in transcription, apoptosis and mitochondrial protein folding (125), while prohibitin 2 is essential in mitochondrial homeostasis and autophagy (126). Du and colleagues identified a protein that has 8 unique peptides matched to human prohibitin, and also shares 40% of sequence similarity, using affinity purification of patient serum and mass spectrometry (127). Seventy-three percent (65/89) of patients with IgG4-RD had antibodies against prohibitin, whilst only 13% with Sjogren’s syndrome and 1.4% of healthy volunteers showed positivity. Moreover, the anti-prohibitin antibody was observed in patients with IgG4-related pancreatic disease, salivary disease, retroperitoneal fibrosis, and other organ involvement (127).

#### 8.1.2 Role of autoantigens as biomarkers in IgG4-RD

Currently, there is no evidence that measuring autoantigens can help to diagnose IgG4-RD, detect response to treatment or predict early signs of a disease flare. There are discrepancies between cohorts on the relative frequency of each autoantigen; Liu and colleagues assessed for the presence of autoantigens in a US IgG4-RD cohort and found 7% positivity for laminin 511-E8, 10% for prohibitin, 12% for annexin A11 and 28% for galectin-3 (118). The authors also observed that there was no clinically meaningful difference between patients with and without the presence of one autoantibody. Nevertheless, patients with positivity for more than one antibody had a more severe presentation, usually with higher levels of inflammatory biomarkers (e.g., total IgG and subclasses, C-reactive protein, and complement consumption) as well as greater risk of visceral organ involvement (118). This is in keeping with findings in other autoimmune diseases.

We can speculate that it is not just one autoantigen that dominates in IgG4-RD, and that the presence of multiple autoantigens may promote a larger breach of B cell tolerance and correlate with a more aggressive inflammatory disease phenotype. Another possible explanation for the wide variety of autoantibodies found in IgG4-RD might be that, in fact, it is not a single disease, but rather a spectrum of disorders characterised by a similar response to different stimuli. Further studies need to demonstrate a single trigger for the disease and/or identify mechanisms to justify why an autoantigen is not involved in all four phenotypes of the disease.

### 8.2 Do patients with IgG4-RD have a biased immune response towards the production of IgG4 against any stimulus?

Another theory behind the development of IgG4-RD is that patients have an inherently biased immune response and preferentially produce IgG4. IgG4 antibody has an important role in the development of tolerance after chronic exposure to allergens (34), and an association between allergy and/or atopy and IgG4-RD has been described by many groups (128–133). There are different definitions for allergy and atopy, which creates a variance in the prevalence of these conditions reported in patients with IgG4-RD (134). Studies observed between 18 and 76% of patients with IgG4-RD have allergy (130–133) and between 14 and 46% have atopy (129, 135, 136). A predominance of type 2 immunity has been reported in such patients. There is an abundance of IL-4 and IL-13, which drive CSR towards IgG4 and IgE production, as well as activation of eosinophils (137). Some studies have suggested a phenotype that is more treatment refractory and aggressive in those with allergy/atopy, higher IgE and IgG4 level and peripheral blood eosinophil counts.

Our group has shown that antigens derived from food and animals elicited a polyclonal IgG4 response in patients with IgG4-RD. These food antigens included egg white and yolk, milk, banana, peanut, rice, wheat, and animal cat dander. Indeed, high serum levels of IgG4 in this setting may reflect a defective regulation of the overall immune response (138).

We have also shown that IgG4-RD may be associated with prolonged contact with occupational antigens. Exposure to solvents, industrial and metal dusts, automotive’s pigments and oils was described in up to 61% of patients in two independent cohorts of IgG4-related sclerosing cholangitis in the Netherlands and the UK (139). Blue collar workers were further identified as a risk factor for developing disease in a case-controlled study (140). No single contaminant was identified, which raises the question whether prolonged contact with a number of environmental antigens trigger the disease and drive the IgG4 response. There is also emerging data on the role of asbestos in IgG4-related retroperitoneal fibrosis, and tobacco smoking conferring an increased risk of developing the disease.

An IgG4 class switch response and production of excessive IgG4 antibodies in the disease is likely determined by the immune cell milieu. During active IgG4-RD, restricted clones of B cells and plasmablasts proliferate through a Tfh2 cell-dependent pathway. Interleukin (IL)-21 producing Tfh2 lymphocytes promote somatic hypermutation in B cells inside GC, while IL-4 producing Tfh2 cells are involved in CSR (141–143). Regulatory T cells and IL-10 are also involved in the making of an IgG4-based response (144, 145).

### 8.3 Are IgG4 antibodies trying to curb an overactive inflammation caused by a different trigger in IgG4-RD?

Finally, we need to consider whether IgG4 antibodies are an epiphenomenon and actually are trying to control inflammation based on a different mechanism. Mouse models confirmed that total IgG from patients with IgG4-RD caused a similar pancreatic injury in the animals (113, 117). Shiokawa and colleagues analysed differences between the pathogenic effect of IgG1 and IgG4 of patients with IgG4-RD when these antibodies were injected in mice (117). When injected separately, both classes caused pancreatic injury and IgG4 also caused salivary damage, but interestingly, when these antibodies were injected simultaneously, IgG4 competed with IgG1 and significantly reduced its binding to pancreatic tissue. Thus, there may be a competitive effect of producing excess IgG4 anitbodies to dampen an inflammatory process that precedes them.

Against this theory is the evidence that IgG4 antibodies in patients with IgG4-RD are highly specific and have high affinity for their target given that circulating B cells are in their majority IgG4^+^ B cells with extensive V-region mutations (91, 92). These findings suggest that these antibodies come from B cells/plasma cells that have undergone rounds of maturation rather than an inflammatory response, thus making the case that IgG4-RD behaves more like an autoimmune disease instead of an inflammatory disorder.

## 9 Parallels between IgG4-AID and IgG4-RD

The antigens proposed for IgG4-RD are ubiquitous and found both in the cytosol and the extra cellular matrix. Nevertheless, with the exception of laminin 511-E8, they fall short of meeting either Witebsky or Koneczny criteria for autoimmunity (61, 146). This brings into question whether these antibodies are the drivers of IgG4-RD. There is evidence that antibodies can penetrate the plasma membrane and recognise intracellular antigens (147–149). In fact, many entry mechanisms have been studied, such as interaction of basic residues with a negatively charged cell membrane (150), Fc-receptor mediated entry (151), endocytosis (147, 152, 153), and nucleoside transporters (154). Among the six IgG4-AID that meet IgG4 autoimmunity criteria, however, all the antigens involved in their pathogenesis are found in the cell membrane or free in the blood circulation (62).

Another important consideration when analysing the role of autoantigens in IgG4-RD is the clinical presentation of the disease. Regardless of phenotype (i.e., pancreatobiliary, head and neck, retroperitoneum and aorta, and systemic), the hallmarks of IgG4-RD are proliferative and/or fibrotic lesions in the organs affected. These findings are diametrically opposed to those from IgG4-AID, where symptoms are driven by IgG4 antibodies blocking antigen function, such as in CNTN1-associated CIDP (155). Histological examination of IgG4-RD lesions shows a massive invasion of IgG4^+^ B cells and plasmablasts and extensive (storiform) fibrosis (156). Furthermore, CD4^+^ and CD8^+^ cytotoxic T lymphocytes are also part of the immune response found in organs with active inflammation and peripheral blood (157), suggesting that the interaction between T and B cells play a critical role in the damage caused by IgG4-RD.

## 10 Current research gaps in IgG4-RD

Despite recent developments in IgG4-RD research, there are essential questions without an answer. One of the most important ones is why and how patients develop it. Previous studies reported an association of HLA-DRB1 and FCGR2B with IgG4-RD, including with phenotypical characteristics (98), and two studies observed a relation between HLA genes, particularly HLA-DQB1*04:01", HLA-DRB1*04:05 and HLA-DRB1*16 in patients with autoimmune pancreatitis type 1 and 2 (158, 159). Moreover, it is also important to look at B cell specialisation and how it can impact humoral immune responses favouring IgG4 expression. As in IgG4-AID, it is paramount to identify whether naturally occurring antigen(s) is(are) driving the immune response in IgG4-RD and the evidence surrounding self-antigens needs to be validated in external cohorts. We must consider the possibility that, such as IgG4-AID, IgG4-RD is a spectrum of diseases rather than a single entity. Finally, the increased expression of IgG4 antibodies and the presence of IgG4^+^ plasma cells in sites with IgG4-RD inflammation highlights the importance of clarifying the mechanisms that favour class switch towards IgG4 rather than other IgG subclasses. This knowledge is crucial to understand the pathogenesis of IgG4-RD (160).

## Author contributions

RVM: Conceptualization, Data curation, Writing – original draft, Writing – review & editing. EC: Conceptualization, Data curation, Supervision, Writing – original draft, Writing – review & editing.
